# pH-Responsive Carboxymethylcellulose Nanoparticles for ^68^Ga-WBC Labeling in PET Imaging

**DOI:** 10.3390/polym11101615

**Published:** 2019-10-05

**Authors:** Anna Maria Piras, Angela Fabiano, Stefania Sartini, Ylenia Zambito, Simona Braccini, Federica Chiellini, Angela G. Cataldi, Francesco Bartoli, Ana de la Fuente, Paola Anna Erba

**Affiliations:** 1 Department of Pharmacy, University of Pisa, Via Bonanno 33, 56126 Pisa, Italy; angela.fabiano@unipi.it (A.F.); stefania.sartini@unipi.it (S.S.); ylenia.zambito@unipi.it (Y.Z.); 2 Department of Chemistry and Industrial Chemistry, UdR INSTM—Pisa, University of Pisa, Via G. Moruzzi 13, 56124 Pisa, Italy; simonabraccini91@gmail.com (S.B.); federica.chiellini@unipi.it (F.C.); 3 Nuclear Medicine, Department of Translational Research and Advanced Technologies in Medicine and Surgery, University of Pisa and Azienda Ospedaliero Universitaria Pisana, 56126 Pisa, Italy; angela.cataldi@for.unipi.it (A.G.C.); francescobartoli1@gmail.com (F.B.); anadf29@hotmail.com (A.d.l.F.); paola.erba@unipi.it (P.A.E.)

**Keywords:** carboxymethylcellulose, nanoparticles, WBC labeling, ^68^Ga, PET, radiolabeling, pH sensitive

## Abstract

Carboxymethylcellulose (CMC) is a well-known pharmaceutical polymer, recently gaining attention in the field of nanomedicine, especially as a polyelectrolyte agent for the formation of complexes with oppositely charged macromolecules. Here, we report on the application of pH-sensitive pharmaceutical grade CMC-based nanoparticles (NP) for white blood cells (WBC) PET imaging. In this context and as an alternative to ^99m^Tc-HMPAO SPECT labeling, the use of ^68^Ga^3+^ as PET radionuclide was investigated since, at early time points, it could provide the greater spatial resolution and patient convenience of PET tomography over SPECT clinical practices. Two operator-friendly kit-type formulations were compared, with the intention of radiolabeling within a short time (10 min), under mild conditions (physiological pH, room temperature) and in agreement with the actual clinically applied guidelines. NP were labeled by directly using ^68^Ga^3+^ eluted in HCL 0.05 N, from hospital suited ^68^Ge/^68^Ga generator and in absence of chelator. The first kit type approach involved the application of ^68^Ga^3+^ as an ionotropic gelation agent for in-situ forming NP. The second kit type approach concerned the re-hydration of a proper freeze-dried injectable NP powder. pH-sensitive NP with 250 nm average diameter and 80% labeling efficacy were obtained. The NP dispersant medium, including a cryoprotective agent, was modulated in order to optimize the Zeta potential value (−18 mV), minimize the NP interaction with serum proteins and guarantee a physiological environment for WBC during NP incubation. Time-dependent WBC radiolabeling was correlated to NP uptake by using both confocal and FT-IR microscopies. The ready to use lyophilized NP formulation approach appears promising as a straightforward ^68^Ga-WBC labeling tool for PET imaging applications.

## 1. Introduction

In recent years, cellulose derivatives have gained renewed interest in the field of green chemistry [1,2] and among those, carboxymethylcellulose (CMC) has immense applications in the food, pharmaceutical, and cosmetic sectors [3]. Pharmaceutical grade sodium CMC is water-soluble at neutral pH, it is available in high purity forms and has found different biomedical applications due to its biocompatibility, rheological properties, and low cost. Given its plant origin, CMC is less likely to cause an immune response, which is a fundamental advantage over other natural additives derived from animals, such as collagen. Furthermore, the digestive enzyme cellulose, cellulase, is absent in humans. This allows for a good in vivo stability, compared to other natural polymeric additives susceptible to enzymatic activity [4]. Thanks to the carboxylic acid functionality, CMC is a polyanion often used as a stabilizer for the synthesis of inorganic nanoparticles [5] and, in the field of polyelectrolyte complexes applications (PEC) [6,7], it is frequently investigated as a key component of three-dimensional hydrogels [8] and microparticles [9].

CMC based nanoparticles (NP) are generally investigated as self-assembled structures from CMC derivatives [10], hybrids or PEC [11]. Presently, the displacement reaction of sodium counterions by other metallic ions has been shown to form coordination complexes, investigated for nanomedicine applications [12]. In this paper, we firstly supposed that this behavior could be exploited for the obtainment of CMC-based NP by ionotropic gelation, in the presence of divalent cations such as Ca^2+^. Secondly, similar ion coordination could be used for the inclusion of Gallium-68 trivalent ion (^68^Ga^3+^), being such a positron-emitting radionuclide for positron emission tomography (PET) imaging. Since 2000, ^68^Ga^3+^ GMP-grade ^68^Ge/^68^Ga generators have been commercialized, thus allowing the in-house production of ^68^Ga-radiolabeled radiopharmaceuticals also in absence of cyclotron facilities. ^68^Ga^3+^ has advantageous decay characteristics (89% β+ yield, 1.9 MeV; half-life of 67.7 min) conferring increasing opportunities for its use in radiopharmacy [13]. In particular, ^68^Ga-PET is thought as a possible alternative for some ^99m^Tc-single photon emission computed tomography (SPECT) applications, due to the better spatial resolution, sensitivity, rapidity of execution, and patient compliance of PET over SPECT imaging [14]. One typical example of such a transition from SPECT- to PET-based radiopharmaceutical is represented by radiolabeled-somatostatin receptor analogs [15].

In the present paper, we focused on the radiolabeling of white blood cells (WBCs) as a model of transition from ^99m^Tc-based to ^68^Ga-radiolabeling, being aware that the potential limitation of ^68^Ga half-life for late-point imaging acquisition. ^99m^Tc-exametazime (^99m^Tc-HMPAO) WBCs radiolabeling is a well-established technique, widely available in all nuclear medicine departments. The radiolabeling procedure is executed by means of devices specifically developed for this application and by the handling of radiopharmaceuticals kits, to guarantee the effectiveness of the procedure along with patient and operator safety. WBCs radiolabeling is performed to detect and localize infections or inflammations and it is applied for the evaluation of several disorders, including osteomyelitis, infected joint and vascular prosthesis, diabetic foot, lung infections, neurological infections, etc. [16,17].

During the last years, nanoparticulate carriers alongside molecular systems, typically bifunctional chelators, have been progressively applied in diagnostic imaging. The main advantage of the use of nanoparticulate carriers regards the possibility of modulating the interaction with the biological system, by tuning the surface and bulk chemical-physical features, and enhancing the efficacy of the transported active, with feasible applications also as theranostic agents [18,19]. In particular, surface features play a major role in mediating the interaction with the biological entities, especially regarding protein interaction and relevant cellular internalization [20,21]. In the case of polymeric NP, the endosomal escape is commonly related to the pH responsiveness of the nanocarrier [22]. In the present studies, we aim to investigate pH-sensitive CMC based NP as radiopharmaceuticals-kit for WBC-labeling. The research activity was performed taking into consideration the need for a simple labeling protocol, quickly executable without risk of potential contamination. Two approaches were pursued, the first involving the simultaneous cross-linking of CMC into NP and ^68^Ga^3+^ loading, readily usable for WBC labeling. The second based on preformed NP, which has to be labeled with ^68^Ga^3+^ under mild conditions, either as NP suspension or lyophilized powder, and then incubated with WBC.

CMC-based NP were prepared by ionotropic gelation and scaled-up. pH-responsiveness, as well as serum stability, was investigated. The optimized formulations were tested for the radiolabeling of freshly isolated WBC under hospital clinical protocols.

## 2. Materials and Methods

### 2.1. Materials

Low viscosity carboxymethylcellulose sodium salt (CMC; Mw = 90,000 g/mol; 0.7 carboxymethyl groups per anhydroglucose unit), Fluoresceienisothiocynate (FITC), ethanol, CaCl_2_ 2H_2_O, Na_2_HPO_4_. 12H_2_O, HCl 1N, NaOH 1N, Trehalose dihydrate; Trizma base, NaCl, and KH_2_PO_4_ were from Sigma (St. Louis, MO, USA). Polyvinylpyrrolidone (PVP-Mw = 25,000 g/mol) was from Carlo Erba (Cornaredo (MI), Italy) and Polyethylenglycol (PEG-Mw = 1500 g/mol) was from Merk Schuchardt (Hohenbrunn, Germany).

### 2.2. Methods

#### 2.2.1. Radioisotope Generation

^68^Ga^3+^ radioisotope was produced by the decay of its radioactive parent Germanium-68 by using a ^68^Ge/^68^Ga generator (Isotope Technologies Garching (itg)). ^68^Ga^3+^ was recovered by low acidic elution according to manufacturer instructions, by using 4 mL of HCl 0.05N. Radioactivity was monitored through dose calibrator apparatus.

#### 2.2.2. Synthesis of Fluoresceinated CMC (FITC-CMC)

2.5% *w*/*v* CMC water solution (1.5 mMols of glucose units) was prepared and pH was adjusted to pH 9 with 1N NaOH. 0.1% FITC solution in DMSO (0.04 mMols) was added to 12 mL of CMC aqueous solution and the reaction was left at room temperature for 20 h. CMC-FITC was double precipitated in ethanol, resulting in an 85 wt % yield.

#### 2.2.3. Sterilization

Glassware sterilization was performed in autoclave (Fedegari Autoclave SPA) at 121 °C, 2 bar for 20 min. Solutions underwent sterile filtration at 0.22 μm (Minisart syringe filter, Sartorius, (Goettingen, Germany) under a laminar flow cabinet.

#### 2.2.4. Diameter Distribution of NP by Dynamic Light Scattering (DLS)

NP diameter distribution was obtained by dynamic light scattering (Zetasizer Nano ZS, Malvern Instruments, Malvern, UK). Samples were analyzed at a 90° angle, at 20 °C and run time of 180 s. Data are reported in terms of average diameter (cumulative intensity evaluation) and polydispersity index (PI).

#### 2.2.5. Attenuated Total Reflectance Infrared Spectroscopy (ATR/FT-IR)

Attenuated total reflectance infrared spectroscopy was performed by using Cary 660 series FTIR, Agilent Technologies, Santa Clara, CA USA. The products are analyzed in a range between 650 and 4000 cm^−1^, with a resolution range of 4 cm^−1^ and 64 scans.

#### 2.2.6. CMC Based NP Formulation by Ionotropic Gelation and Kit-Type I Formulation

CMC based NP formulations were investigated as reported in Table 1 in the absence of radionuclide, the optimized formulation (Run 15) was prepared as follows. CMC was dissolved in deionized water, overnight, to a final concentration of 0.6 wt %, in 2 mL volume. The CMC solution was completed by adding 50 μL of Phosphate buffer (PB 0.4 M pH 8.5) and 210 μL of NaOH 0.1 N, and 0.22 μm filtered under a laminar flow cabinet. The obtained CMC solution, having pH ≈ 11, was closed into a sterilized vial. A water solution of CaCl_2_, 10 wt%, was prepared, 0.22 μm filtered under a laminar flow cabinet and closed into a sterilized vial. For the preparation of the NP, 3.5 mL of HCl 0.05 N (simulating the elution medium of the radionuclide), were added to the CMC solution, followed by the addition of 0.8 mL of the CaCl_2_ solution. Ten minutes were necessary to consolidate the NP in suspension, having a pH of 7.4. Concerning the first kit-type approach (In-situNP), i.e., the preparation of NP at the site of use, a similar procedure was followed but instead of plain 0.05 N HCl, the freshly eluted ^68^Ga solution was used, under laminar flow cabinet.

#### 2.2.7. Kit-Type II Formulations

Two formulation approaches were investigated, namely the use of a purified NP suspension (NPSusp) and a purified NP powder obtained by lyophilization (NPLyo). In both cases, NP formulation RUN 14 was scaled up 30 times (RUN 16) and the obtained NP suspension was purified by centrifugation 6000 rpm × 20 min, 4 °C (Sorvall, MTX, micro-ultracentrifuge, Thermoscientific, Waltham, MA, USA). NP pellet was re-dispersed in 0.4 mM CaCl_2_ solution in 0.1 M Tris buffer (pH 7.4). The obtained suspension was named NPSusp.

The lyophilized powder NPLyo was prepared starting from NPSusp, by adding trehalose to a final 5 wt % concentration. Lyphylization (VirTis AdVantage wizard 2.0, SP Scientific, Stone Ridge, NY, USA) was performed starting from a freezing temperature of −40°C. For FITC-labeled NP (FITC-NPlyo), NP were prepared by using 15 wt% of FITC-CMC.

#### 2.2.8. Effect of Cryoprotecting Agents on NP Lyophilization

CMC-based NP suspension (NPSusp) was used for the setup of the lyophilization conditions. Three cryoprotecting agents were tested, namely trehalose, PEG and PVP in concentrations between 1 wt % and 5 wt %, and at freezing temperatures of −20 °C or −40 °C (Table 4). Once lyophilized, the powders were re-dispersed in 0.05 N HCl, mimicking the radionuclide solution, and the diameter distribution of the recovered NP was determined by DLS measurements.

#### 2.2.9. Radiolabeling of Preformed CMC Based NP

NPSusp underwent labeling studies in the presence of ^68^GaCl_3_ 0.05 N HCl solution, in order to assess the optimal conditions in terms of concentration and incubation time. Five NP concentrations in the range of 1–5 mg/mL were placed in the presence of ^68^Ga^3+^ with a 1114 Ci/mL radioactivity, under a laminar flow cabinet. The suspensions were incubated either for 5 min or for 15 min and centrifuged at 14,000 rpm for 10 min (Hettich Zentrifugen Mikro 120, Tuttlingen, Germany). Both NP pellet and supernatant radioactivity were measured (dose calibrator) and used for the calculation of specific radioactivity (RADSt μCi/mg) and labeling efficiency (LE%) as follows:

(1) $$RADSt=\frac{Radioactivity\;of\;NP\;Pellet}{\;NP\;pellet\;dry\;weight}$$

(2) $$LE\%=\frac{Radioactivity\;of\;NP\;Pellet\;}{Radioactivity\;of\;NP\;Pellet+\;Radioactivity\;of\;supernatant}\;\times 100$$

#### 2.2.10. Evaluation of NP pH Sensitivity

CMC-based NP (RUN16) were purified by centrifugation and the pellet was re-dispersed in 10 mL of Tris 0.1 M pH7.5. The solution pH was stepwise lowered by using HCl 1 N. DLS measurements were performed after 5 min of equilibrating time (Zetasizer Nano ZS, Malvern Instruments, Malvern, UK).

#### 2.2.11. NP Stability in the Presence of Serum Proteins

Purified NP (RUN 16) were re-dispersed in 0.1 M Tris-buffered (TB) at pH 7.4 to a final concentration of 5 mg/mL of NP. The suspension was aliquoted and added with CaCl_2_, NaCl, and trehalose in order to investigate the effect of medium composition on NP stability. Six different media were assayed: TB (Plain), 0.4 mM CaCl_2_ in TB (CaCl_2_), 0.9% NaCl in TB (NaCl), 0.4 mM CaCl_2_ and 0.9% NaCl in TB (CaCl_2_ and NaCl), 5% Trehalose in TB (Trehalose), and 0.4 mM CaCl_2_ 5% Trehalose in TB (Trehalose and CaCl_2_).

The obtained NP suspensions underwent Zeta potential (ζ) evaluation. Measurements were performed by using DelsaTM Nano C (Beckman Coulter, Indianapolis, IN, USA), at 25 °C, in low concentration cell.

The same samples were added with 5% *v*/*v* cell-free plasma (CFP), collected from fresh blood during the WBC isolation procedure. The incubation period occurring for the formation of aggregates (coagula) was timed and evaluated by visual inspection [16].

#### 2.2.12. Isolation of Mixed Human Leukocytes and Labeling

Mixed WBCs (1.8 × 108 cells) were freshly isolated by following erythrocyte sedimentation, from venous whole blood (40 mL) taken from volunteers. The procedure was performed according to standard hospital guidelines [16] by using Leukokit (Gipharma), and by following producer instructions. The cellular pellet was double washed with PBS and afterwards incubated with 5 mL of radiolabeled NP at room temperature. At pre-established times, the mixture was centrifuged 150 gx5 min (Centrifuge Eppendorf 5804 R) and radioactivity of cellular pellet and supernatant was measured (Dose calibrator). Radioactivity internalization efficiency (INT%) is expressed as the percentage of the ratio between cellular pellet radioactivity and total radioactivity. To assess the retention of radioactivity, 45 min labeled WBC were re-dispersed in PBS pH7.4 and incubated for 15 min and 45 min. At the end of these incubation times, WBC were isolated by centrifugation and radioactivity was counted. The permanence of radioactivity within WBC (RES%) was expressed as the percentage of the ratio between cellular radioactivity and total internalized radioactivity. Cell viability was monitored by trypan blue exclusion assay [16].

#### 2.2.13. Internalization of Fluorescein Labeled NPlyo and Micro FT-IR Analysis

Freshly isolated WBC pellet (2 × 108 cells) was washed two times with PBS (4 mL) and incubated with 4 mL of FITC-NPlyo (5 mg/mL re-dispersed in HCl 0.01 N). Every 15 min, three aliquots were collected and WBC were recovered by centrifugation (900 rpm × 5 min). For confocal imaging, pellets were re-dispersed in PBS, fixed on microscope coverslip glasses (∅ 16 mm) coated with 0.01% poly-l-lysine solution (Sigma Aldrich, St. Louis, MO, USA) and analyzed using a Nikon Eclipse TE2000 inverted microscope equipped with EZ-C1 confocal laser (Nikon, Tokyo, Japan) apparatus. An argon ion laser (488 nm emission) was used to excite FITC fluorophore. Images were captured with Nikon EZ-C1 software with identical settings for each sample.

For FT-IR imaging, WBC pellets were incubated for 10 min in 4% paraformaldehyde, then washed two times with deionized water, fixed on corner frosted Low-e microscope slides (Kevley Technologies) vacuum dried and stored under vacuum until measurements. FTIR spectroscopic imaging was conducted using an Agilent Cary 620 microscope coupled to the Cary 660 spectrometer, (Agilent Technologies), with a focal plane array (FPA) detector, under reflection mode, cooled by liquid nitrogen, 0.62 as numerical aperture, and magnification 15×. Spectra were collected at 4 cm^−1^ resolution, pixel size of 5.5 μm, with 64 scans and 3500–800 cm^−1^ spectral width, applying triangular apodization after spectra collection. The second derivatives of the acquired spectra were calculated after Savitzky-Golay 9 points smoothing. Post-processing included the average of 10 selected spectra per field and the comparison of band intensity after normalization on amide I band (1640 cm^−1^).

All experiments were performed on at least three replicates. The experiments were expressed as the mean and standard error of mean (SEM) values for each group.

## 3. Results and Discussion

In the field of biomedical imaging, positron emission tomography (PET), with its ability to identify and quantify picomolar quantities of radionuclide, has emerged as one of the most powerful diagnostic techniques. Currently, studies concerning polymeric vehicles for ^68^Ga radiolabeling are relatively few and involve hybrid systems such as coated inorganic nanoparticles, methacrylate polymers or dendrimers (i.e., PAMAM) [23,24]. The choice of CMC for this specific ^68^Ga-NP application stems from the desire to work with a known product already in pharmaceutical use. This choice could facilitate a faster clinical translation of the performed studies. Additionally, despite being known and used for decades as a rheological excipient for numerous pharmaceutical applications, CMC transformation into nanoparticles by ionotropic cross-linking can equally have interesting scenarios and, to our knowledge, has not been extensively explored, yet. In the present research work, it was possible to prepare two CMC-based nanoparticulate systems: In the first case the trivalent ion nature of ^68^Ga^3+^ was used as co-crosslinker for ionotropic gelation, in the second, instead, the labeling was performed of preformed particles.

### 3.1. Radiolabeling and In-Situ Formation of CMC Based NP: I KIT-Type Approach

In this first approach, the radionuclide eluted in 0.05N HCL solution is added directly to CMC solutions for the formation of NP by ionotropic gelation, in the presence of Ca^2+^ as a cross-linking agent. Initially, the optimization of the formulation parameters was related to the formation of an opalescent suspension of NP with uniform diameter distribution and an average diameter below 500 nm. Furthermore, it was necessary to obtain NP suspension suitable for direct incubation with living leukocytes: That means sterility, neutral pH, and isotonicity. Additionally, fast incubation and operator-friendly procedures had been considered. The simple handling and mixing of three solutions were envisaged: A solution of CMC, the acidic freshly eluted solution of ^68^GaCl_3,_ and a solution of CaCl_2_ as a consolidating agent. During the preliminary formulation studies, a non-radioactive GaCl_3_ in 0.05 N HCl solution was used.

As reported in Table 1, several combinations of CMC/CaCl_2_wt ratios were assayed, as well as the use of a buffered solution. Initially, it was observed that NP formation was not occurring in the presence of the sole ^68^GaCl_3_ solution (RUN 1 and 2) due to the extremely low concentration of salt (0.003 nM), and that NP formation was sensitive to the solution pH as expected (RUN 10, 11). For the tuning of the final pH value, the combination of PB and NaOH 1 N was examined considering also that the addition of CaCl_2_ lowered the pH and that stronger PB was altering the tonicity of the solution as well as leading to the formation of calcium phosphate precipitates. The formulation named RUN 15, resulted perfectly buffered without any additional pH adjustment and leading to a homogeneous diameter distribution with a mean diameter of 200 nm. The ionotropic cross-linking process is a cooperative process [25] and requires a consolidation time to stabilize the cross-linked macromolecules into NP. Being in the presence of a radionuclide, rapid consolidation time reduces the loss of radioactivity due to the normal radioactivity decay and it is preferable for operator exposure safety. The optimal consolidation time was found to be 10 min from the addition of CaCl_2_ solution. Additional time was not significantly modifying, either the diameter distribution, or the scattering intensity (i.e., 5 × 10^−6^ counts/s).

**Figure 1:**
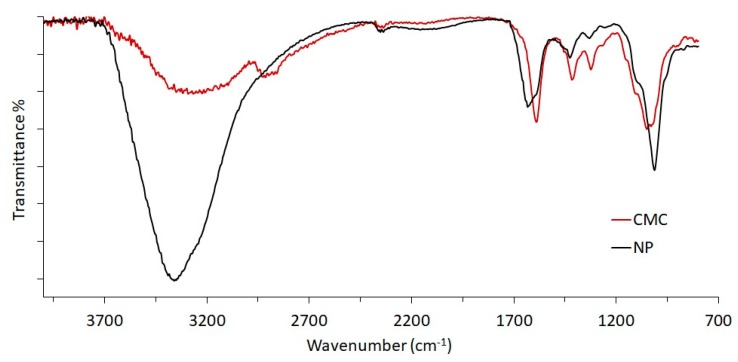
ATR/FT-IR spectra of pristine CMC and purified NP from In-situNP sample.

The ionotropic cross-linking of CMC into NP was confirmed by ATR/FT-IR spectroscopy (Figure 1). The comparison of native CMC and NP evidenced the shifting of the O–H band from 3300 nm^−1^ to 3359 nm^−1^, including an increased intensity and narrowed amplitude. Furthermore, the carboxylate bands at 1589 and 1416 nm^−1^ for the CMC spectrum shifted to 1632 and 1426 nm^−1^. These differences are assigned to the presence of calcium ions acting as bridges between two carboxylate moieties, thus cross-linking the CMC. Consequently, the free carboxylate moieties are reduced in number, determining also a variation of the intra-/intermolecular hydrogen bridges between carboxyl and backbone hydroxyls.

The selected formulation was then prepared in the presence of freshly eluted ^68^Ga^3+^ and characterized. Filter sterilized solutions of CMC and CaCl_2_ were placed in autoclaved glass bottles and sealed, before being submitted to ^68^Ga^3+^ labeling/NP formation. No substantial differences were observed, in terms of size distribution, yield, and final pH, between the suspensions prepared in the simulated medium (RUN 15) and those prepared in the presence of the radioactive (named In-situNP, Table 2). The specific radioactivity measured on centrifuged NP resulted in 156 mCi/mg, but the LE% was 11.4.

### 3.2. Radiolabeling of Preformed CMC NP: II KIT-Type

The second modality investigated for the labeling of NP of CMC concerns the use of a preformed NP sample, to be incubated with the freshly eluted radioactive agent. Similarly, the essential requirements were a homogeneous nanometer-scale diameter distribution, physiological pH and isotonicity of the resulting suspension, rapidity, and simplicity of handling for the operator, good specific radioactivity of the resulting NP. Two approaches were investigated: A concentrated, purified NP suspension (NPSusp) and a lyophilized NP powder (NPLyo). In both cases, it was first necessary to scale up the NP preparation protocol up to 30 times the original RUN14 formulation scheme. The obtained NP suspension (RUN16) have an average diameter of 217 nm with a low polydispersity index (PI: 0.333), in agreement with RUN14 results (Table 1). NP purification was performed by centrifugation and the conditions were optimized in order to maximize the yield, resulting as 31.4 ± 0.6 wt %, but preserving a nanoscaled diameter distribution (**Ø** of purified NP: 296.4 ± 6.3 nm, PI 0.323).

**Figure 2:**
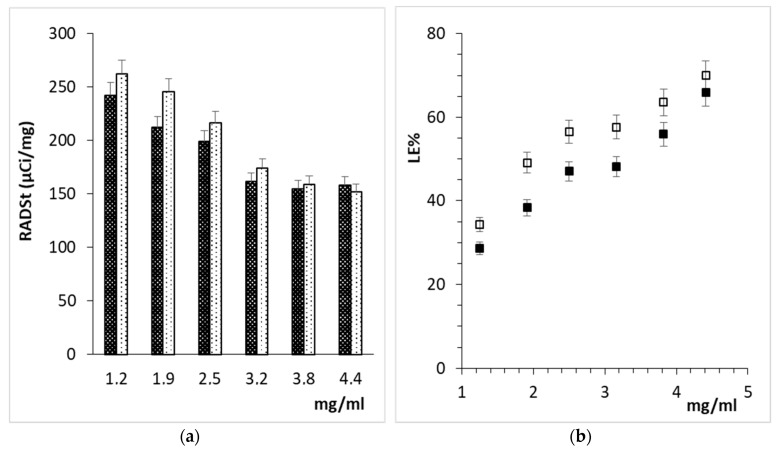
Effect of concentration and incubation time on NPSusp radiolabeling: (**a**) RADst after 5 (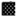) and 15 min (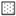), (**b**) LE% after 5 min (■) or 15 min (□).

The labeling of NPSusp in terms of RADst and LE% (Figure 2) was investigated at two different incubation times and by varying NP concentration in solution. Lower NP concentrations displayed higher RADst but lower LE%, in agreement with the dynamics of the supposed complexing/Ca^2+^-exchange with CMC carboxylate moieties. The variation of the incubation time from 5 min to 15 min did not improve RADst due to concomitant ^68^Ga^3+^ radioactivity decay. A concentration of 5 mg/mL was selected and applied for the subsequent investigations.

Lyophilization conditions for purified NP were also assayed. Several conventional cryoprotective agents were tested, such as PVP, PEG, and trehalose. The lyophilized samples were then re-dispersed in 0.05 N HCl (mimicking the elution buffer of the radioisotope), and analyzed by DLS. Among tested conditions, 5% trehalose was the most effective cryoprotecting agent and allowed for preserving the nanoscaled diameter distribution (Table 3). Diversely, the other agents caused a considerable increase in the polydispersion and significant variations in the average diameter value. The physiological pH value of the re-dispersed NPLyo was guaranteed by using Tris base buffer (0.1 M). The organic buffer was preferred over the more common phosphate buffer, thanks to its poor contribution to the solution osmolarity [26] and to avoid the salting-out effect, otherwise induced by PB during the freeze-drying process.

The radiolabeling of preformed CMC-based NP (the II kit type approach) involved the preparation of both NPSusp and NPLyo by using filter-sterilized solutions, and the execution of the protocol under a laminar flow cabinet. Freshly generated ^68^Ga^3+^ solution was applied, and Table 2 displays the labeling results of NPSusp and NPLyo. Both formulations led to LE% higher than 80% and comparable RADst. The higher RADst values recorded for NPsusp are ascribable to the wider specific surface of the smaller NP of NPSusp (average diameter of 299 vs. 343 nm of NPLyo).

### 3.3. NP Stability: Effects of pH Acidification and Serum

**Figure 3:**
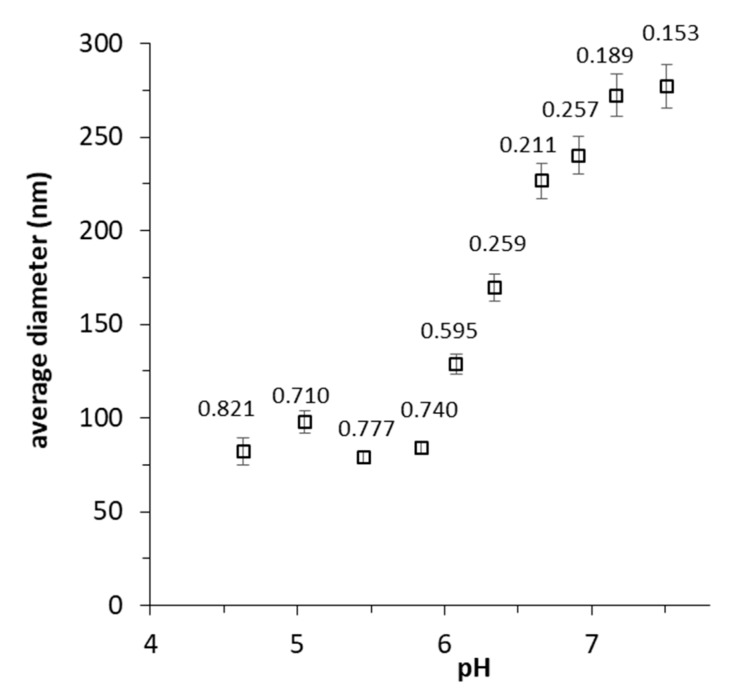
Plot of DLS average diameter vs. solution pH. There is a clear trend of NP diameter reduction with pH acidification. Labels over the marks refer to the polydispersity index (PI) of the diameter distribution.

The response of purified NP to pH variation was assessed by DLS measurements. The suspension remained opalescent, without significant variation in the size distribution until pH 6.5. Lower pHs corresponded to the loss of stability, with a gradual reduction of NP average diameter, combined with a massive increase of polydispersity (Figure 3). This behavior is indicative of the loss of cross-linking due to the increasing of carboxylic moieties with respect to carboxylate ones, as approaching polymer pKa (CMC pKa: 4.30). Meanwhile, the loss of solubility of CMC at lower pHs was reflected by the formation of micron-sized aggregates (Ø > 4 μm), with sedimentation tendency.

NP stability in serum is a central reason for their application as WBC-labeling agent. Tris-buffered 5 mg/mL NP suspensions (named as Plain) were used and added either with NaCl under physiological osmolarity concentration or trehalose. CaCl_2_ was also added to 0.42 mM final concentration, as typically adopted in cell culturing media suited for WBC [27], such as the Roswell Park Memorial Institute (RPMI)-1640. The assayed samples represent the potential media in which the WBC internalization tests can be carried out, considering freshly isolated WBC from peripheral blood. The presence of calcium allows for the mediation of the endocytosis process, while sodium chloride and trehalose guarantee an isotonic environment and, for the latter, the efficacy of the lyophilization process.

**Figure 4:**
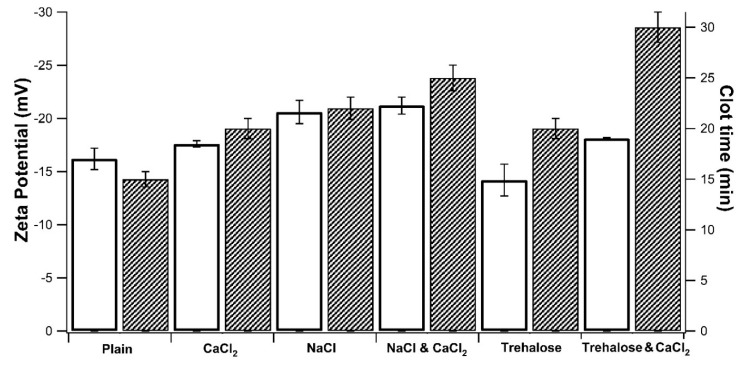
Effect of medium composition on NP zeta potential value (□) and time of clot formation in the presence of serum proteins (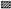). All samples contained CMC-based NP (5 mg/mL) in 0.1 M Tris-buffered at pH 7.4 (Plain), then, salts and cryo-protector agent were added either alone or combined: CaCl_2_ (0.4 mM), NaCl (0.9%), and Trehalose (5%).

The interaction study with serum was mandatory for the selection of the best incubation conditions, since CMC can directly interact with the residual serum proteins entrapped in wet WBC isolated pellets, forming with those aberrant coagula which leads to procedure failing. NP stability in serum was correlated with the time needed for the formation of macroscopic clots in the dispersing media, containing 5% *v*/*v* of CPF [16]. The samples were also characterized in terms of Zeta potential (ζ), measured in the absence of CPF. The recorded data are plotted in Figure 4 and a strict correlation was observed between NP stability with serum and the corresponding ζ value. All the zeta potential values were found to be negative, due to the carboxylate moieties of CMC. Taking the plain samples as a reference (ζ −17.2 mV), the absolute value always increased with the addition of inorganic salts. Differently, in the presence of trehalose the absolute ζ value decreased, remaining negative, anyway. Trehalose is a non-ionic disaccharide, therefore, it does not act as a counter-ion within the electrical double layer on the NP surface, but its effect is more easily ascribable to non-specific adsorption. Hence, there is a screening action toward the surface charges and a consequent reduction of the absolute ζ value. Although further investigations are necessary, it is possible to state that the extension of stability timing in serum is strictly linked to the surface properties of NP in suspension. The screening effect of more stable NP with high ζ potential values, as well as adsorption of trehalose on the colloid surface, resulted in a delayed coagula formation.

To summarize, the pH sensitivity of the CMC-based NP and the tendency of CMC to interact with serum proteins were confirmed. The optimization of the formulation aspects was necessary to limit the formation of coagula during the subsequent studies of cellular uptake. However, both aspects appear useful and promising for the modulation of the intracellular trafficking of CMC based NP. In the present application, the pH-sensitivity can be useful for the endosomal escape by exploiting the natural pH acidification of endosome maturation, thus leading to a reduced radioactivity efflux for radionuclide exchange through cellular proteins interaction.

### 3.4. WBC Labeling

The two kit type approaches for WBC radiolabeling were compared through the WBC internalization assessment. The In-situNP formulation was tested, involving the concomitant formation of NP suspension and labeling guided by the ionotropic gelation mechanism. The second approach, concerning the labeling of preformed NP, was investigated for both NPSusp and NPLyo formulations. The WBC internalization tests were carried out by using WBC isolated from donor peripheral blood. The internalization was monitored for incubation periods up to 45 min and was evaluated in terms of radioactivity recovered in WBC (INT%). The permanence of the radioactivity (RES%) in labeled isolated WBC was also measured, suggesting partial radioactivity outflow over time (Table 4).

Concerning In-situNP, I procedure approach, INT% is low and it was not time-dependent. Indeed, In-situNP presented a large amount of free CMC, compared to that truly converted to NP (33 wt %), which was not significantly internalized by WBC giving no contribution to cell labeling. Additionally, it was not possible to calculate the outflow due to the low residual radioactivity in cell pellets, which was not enough for a reliable reading.

Regarding the preformed NP, II procedure approach, both NPSusp and NPLyo were tested. NPSusp excipients were Tris buffer, CaCl_2,_ and NaCl, similarly to NPLyo, except for the switch of NaCl with trehalose. INT% was measured at 15, 30, and 45 min but NPSup failed visual inspection after 20 min of incubation, due to the formation of macroscopic aggregates and indicating non-sufficient stability in the presence of the cellular pellet. Considering the specific hospital guidelines [16], the formation of precipitates compromises the successful use of labeled-WBC suspension, being unsuited for patient re-infusion.

Conversely, NPLyo passed both visual inspection and trypan blue exclusion test [16] with less than 1% of cell damage during the entire study (efflux period included). The internalization data display increasing radioactivity over time, suggesting for time-dependent cellular uptake. The samples incubated for 45 min were submitted to the efflux study, monitoring the radioactivity of WBC after 15 and 45 min. A reduction of RES% over time is observed, following radioactive outflow. The residual percentage radioactivity at 45 min was equal to 52%. The assessment of longer periods was not performed because of the rapid radioactivity decay of ^68^Ga (t_1/2_: 68 min) and considering that at least 90 min (sum of 45 min incubation time and 45 min for efflux evaluation) passed since ^68^Ga had been eluted from the generator.

Indeed, the surface characteristics of the nanoparticle (charge density, presence of hydrophobic domains, stealth molecules or directional molecules) and the cellular environment are very important and correlated factors [28]. Typically, cationic nanoparticles carry a better interaction with the negatively charged cell membrane. On the other hand, the opsonization process generally mediates the uptake of negatively charged NP, with a significant contribution depending on the type of adsorbed protein. It is interesting to note that even the protocol applied to cell isolation can affect the mechanisms of NP uptake. Baumann et al. (2013) reported that, in the case of negatively charged NP, the uptake from leukocytes can strictly depend on the anticoagulant type and that heparin can significantly increase the internalization compared to those positively charged NP. In the present work, an interaction between NP and serum was observed, leading to the formation of macroscopic coagula. However, the working conditions were optimized and included both cell pellet washing and the presence of agents capable of slowing down the interaction with serum, allowing for the selection of the NPLyo protocol as the best candidate for WBC-labeling.

### 3.5. Assessment of NP Uptake from WBC

**Figure 5:**
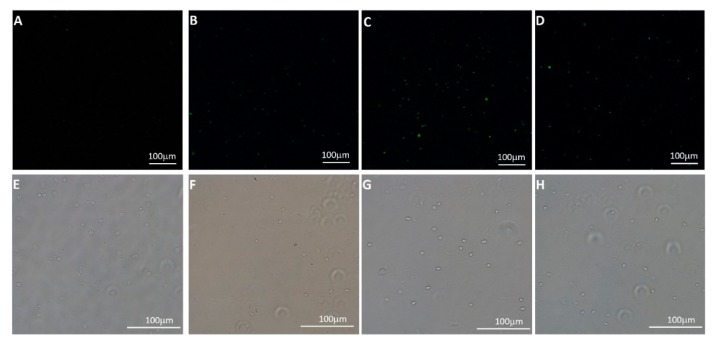
Micrographs of FITC-NPLyo labeled WBC after different incubation periods: control (**A**,**E**), 15 min (**B**,**F**), 30 min (**C**,**D**), 45 min (**D**,**H**). Fluorescence images 20× magnification (**A**–**D**), and photo-micrograph of the sampled region, acquired using a brightfield camera with a 20× magnification (**E**–**H**). Scale bars correspond to 100 μm.

In order to confirm that the acquisition of radioactivity from WBC is due to NP uptake from cells and not to non-specific labeling, FITC labeled NPLyo (FITC-NPLyo) were prepared. The nanoparticles had similar physical-chemical features to the pristine NPLyo formulation, with an average diameter of 238.7 ± 5.0 nm, PI of 0.352 and ζ potential value of −21.1 ± 0.3 mV. The WBC-labeling (II approach procedure) was applied to FITC-NPLyo, and freshly isolated WBC were incubated with the particles. Confocal microscopy images were acquired on isolated and purified cells after incubation with FITC-NPLyo, indicating that the detected fluorescence is ascribable to uptaken particles (Figure 5). Additionally, the intensity and number of fluorescence spots increased with longer incubation times, in agreement with what already observed in terms of radioactivity for gallium labeled WBC by means of NPLyo and confirming the specific radiolabeling due to NP uptake.

Since it is known that the size of NP greatly influences the speed of internalization and, although this speed also depends on the type of cell considered, nanoparticles with an average diameter of less than 100 nm generally have higher uptake rates than nanoparticles with diameters between 100 and 500 nm [28]. For NP with 200–300 nm average diameter, similarly to the investigated CMC-based NP, the uptake studies are generally performed with longer incubation times, at least 1 h, and kinetics evaluations up to 8 or 24 h [29]. Additionally, the uptake of NPLyo was time-dependent even in short time (up to 45 min), and such timing restrictions were necessary because of the fast ^68^Ga^3+^ radioactivity decay.

**Figure 6:**
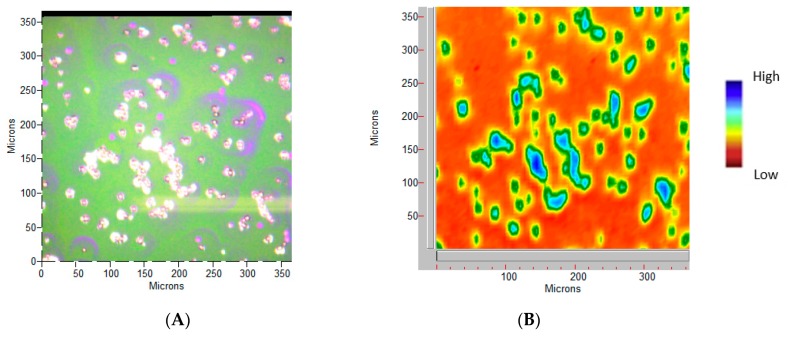
WBC NPLyo 30 min labeled sample. (**A**) Photo-micrograph at 15× magnification, at a pixel resolution of 5.5 μm. (**B**) The Amide I integrated peak area map, in false colors.

FT-IR imaging can probe the cellular macromolecular components through their fingerprint characteristic bands [30]. The main characteristic bands attributed to proteins, lipids, DNA, and polysaccharides [31] of WCB are summarized in Table 5, whereas NPLyo labeled WBC morphology and related vibrational mapping (set on ~1620 cm^−1^ band, Amide I) are displayed in Figure 6.

**Figure 7:**
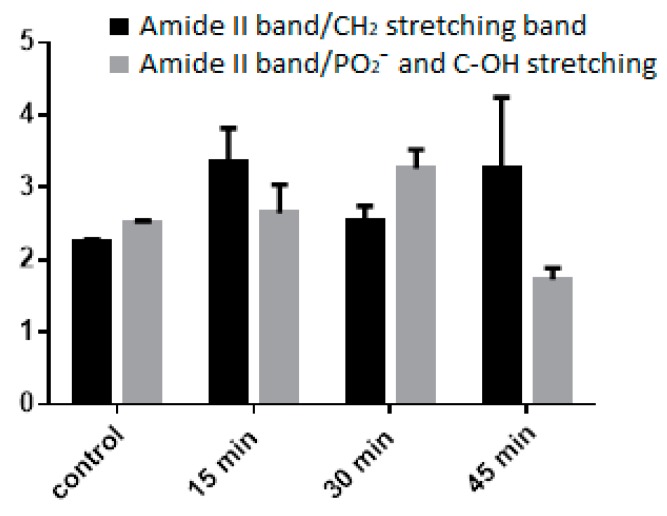
Area ratio of macromolecular main components. Reference band: Amide II (1533 cm^−1^).

As shown in Figure 7, the NPLyo labeled WBC preserve the main macromolecular composition during incubation. The reduction of the 45 min ratio may be attributed to the increased concentration of saccharides, related to the uptaking of the CMC-based NP. Similar analyses have been recently proposed to collect the spectral mapping of disease states in tissues or assess the in vitro drug-related cellular toxicity [31]. Thus, the obtained results suggest a non-cytotoxicity effect of NPLyo, as already seen by trypan blue exclusion test, and for a preserved functionality of WBC.

## 4. Conclusions

This is the first report on the possible application of pharmaceutical grade CMC-based NP as a kit-type component for WBC-radiolabeling. Both investigated approaches were practically applicable and adaptable to the current guidelines followed for ^99m^Tc-HMPAO WBC-labeling. The lyophilized powder approach is thought to be preferred, due to both handling aspects of labeling protocol and longer storage stability. In vitro investigations have confirmed the labeling of WBC due to NP uptake, without altering cell vitality.

NP marking was managed under mild conditions, using the acidic ^68^Ga^3+^ 0.05 N HCl solution, freshly eluted on-site by the ^68^Ge/^68^Ga generator. The direct use of the elution solution opens to the future possibility of transferring the investigated CMC-based NP to PET/CT operating also with longer-living radiopharmaceuticals, such as ^44^Sc, more suitable for the whole assessment of the WBC kinetic over time. Actually, ^44^Sc has a longer half-time (3.92 h) and can be produced either by ^44^Ti/^44^Sc generator or by small cyclotrons, in both cases by acidic HCl solutions [32]. Additionally, in order to accelerate WBC-labeling rate, the reduction of NP average diameter to less than 100 nm by upgrading to microfluidics apparatus [33] or electrodynamic atomization [34], and the covalent binding of specific chelators [13] might be proposed.

## Figures and Tables

**Table 1:** In-situ nanoparticles (NP) formulation parameters, final suspension pH and diameter distribution (DLS).

RUN	CMC		PB 0.4 M	NaOH	HCl 0.05 N	CaCl_2_ 10%	CMC/	pH	Ø Distribution	
	(mL)	wt%	(uL)	0.1 N (uL)	(mL)	(mL)	CaCl_2_		nm± SD	PI
**1**	2	1.5	-	-	4.0 ^a^	-	-	-	-	-
**2**	2	0.6	50	115	2.0 ^a^	-	-	-		
**3**	3	1.5	-	-	-	1	0.5	-	-	-
**4**	1	4	125	-	1.0	0.2	2.0	-	-	-
**5**	1	4	125	-	1.0	0.4	1.0	-	-	-
**6**	2	1.5	180	-	4.0	1	0.3	5.3	-	-
**7**	2	1.5	500	-	4.0	1	0.3	5.3	-	-
**8**	2	4	250	-	2.0	0.4	2.0	5.6	-	-
**9**	2	1.2	50	80	2.0	0.41	0.6	5.7	-	-
**10**	2	4	150	40	2.0	0.4	2.0	5.8	-	-
**11**	2	1.2	50	90	2.0	0.4	0.6	6.8	394 ± 2.3	0.518
**12**	2	4	150	70	2.0	0.4	2.0	6.9	284 ± 9.6	0.560
**13**	2	4	50	100	2.0	0.4	2.0	7.4	267 ± 6.3	1.054
**14**	2	0.6	50	115	2.0	0.7	0.2	7.4 ^b^	221 ± 3.9	0.313
**15**	2	0.6	50	210	3.5	0.8	0.2	7.4	203 ± 5.1	0.323
**16**	60	0.6	1500	3450	60.0	21.0	0.2	7.4 ^b^	217 ± 3.3	0.333

^a^ GaCl_3_ solution; ^b^ pH adjusted by adding NaOH 0.1 N, after particle consolidation period.

**Table 2:** Main Characteristics of the NP sample as resulting from the two kit-type approaches.

Kit Type Approach	NP	Ø Distribution nm ± SD	PI	RADSt (mCi/mg)	LE %
***1***	**In-situNP**	206.0 ± 6.7	0.312	156 ± 7.8	11.4
**2**	**NPSusp**	299.5 ± 4.3	0.336	222 ± 11.1	81
	**NPLyo**	342.4 ± 5.1 ^a^	0.332	175 ± 8.7	83

^a^ Diameter distribution of NPLyo re-dispersed in ^68^Ga 0.05 N HCl.

**Table 3:** Optimization of freezing temperature and cryoprotectant concentration, for NPLyo lyophilization.

Cryoprotectant		Freezing	Ø Distribution	
Agent	wt %	°C	nm ± SD	PI
Trehalose	5	−20	615.6 ± 286.0	0.468
PVP	3	−20	152.6 ± 10.60	1.808
PVP ^a^	1	−20	-	-
PEG	3	−20	1147.4 ± 168.0	1.384
Trehalose	5	−40	345 ± 8.20	0.323
PVP	3	−40	750.5 ± 24.95	0.851
PVP ^a^	1	−40	-	-
PEG	3	−40	447.4 ± 11.15	0.336

^a^ In the presence of PVP, macroscopic aggregates were formed and precipitated; DLS was not run.

**Table 4:** White blood cells (WBC) labeling efficiency (INT%) and retention of radioactivity over time (RES%).

Sample	INT%			RES%	
	15 min	30 min	45 min	15 min	45 min
**In-situNP**	3.2 ± 0.7	3.3 ± 0.3	3.3 ± 0.9	-	-
**NPSusp**	9.3 ± 0.3	15.6 ± 1.3	17.0 ± 0.7	-	-
**NPLyo**	8 ± 0.4	13.5 ± 0.3	16.1 ± 0.9	63 ± 1.2	52 ± 1.2

**Table 5:** Assignment of macromolecular relevant bands used in this work.

FTIR Peak (cm^−1^)	Assignment
2928	Asymmetric stretching CH_2_
2860	Stretching of lipids
1637	C=O stretching of amide protein (Amide I)
1533	N–H bending of amide protein (Amide II)
1228	Asymmetric stretching of nucleic acids PO_2_^−^
1075	Overlapping of symmetric stretching of nucleic acids PO_2_^−^ and C–OH Stretching of oligosaccharides
